# Equity Implications of Hospital Penalties During 4 Years of the Comprehensive Care for Joint Replacement Model, 2016 to 2019

**DOI:** 10.1001/jamahealthforum.2022.4455

**Published:** 2022-12-02

**Authors:** Sukruth A. Shashikumar, Andrew M. Ryan, Karen E. Joynt Maddox

**Affiliations:** 1 Cardiovascular Division, Department of Medicine, Washington University School of Medicine in St Louis, St Louis, Missouri; 2 School of Public Health, Brown University, Providence, Rhode Island; 3 Center for Health Economics and Policy, Institute for Public Health, Washington University in St Louis, St Louis, Missouri

## Abstract

This cross-sectional study assesses the penalties against hospitals under the Comprehensive Care for Joint Replacement model mandated by Medicare, with particular attention to safety-net hospitals and those serving a high proportion of Black or Hispanic patients.

## Introduction

Comprehensive Care for Joint Replacement (CJR) is a mandatory Medicare bundled payment model in which hospitals receive a target spending price for joint replacement episodes spanning from admission through 90 days after discharge. Hospitals that reduce spending below their target receive a bonus; those that fail to do so are financially penalized.

From 2016 to 2019, CJR incrementally shifted target prices from hospital-specific targets to regional targets.^1^ Although participation was initially mandatory for hospitals in 67 randomly selected markets, only hospitals in the 34 highest cost markets were mandated to participate after 2017.^1^ Little is known about how Medicare distributed penalties under these policy changes. Given the disproportionate penalization of safety-net hospitals and hospitals serving Black and Hispanic patients under value-based payment,^2,3^ understanding trends in their penalization status under CJR has important equity implications as Medicare expands bundled payment.

## Methods

We obtained CJR performance information from Medicare data^1^ and hospital characteristics from 2016 to 2019 Impact Files, 2017 inpatient claims, and the 2018 American Hospital Association Survey. Hospitals without data were excluded (15 in 2016, 16 in 2017, 6 in 2018, and 1 in 2019). Hospitals with high Black and Hispanic populations were defined as those in the top quintile of proportion of patients of Black and Hispanic race and ethnicity. Safety-net hospitals were defined as those in the top quintile of Disproportionate Share Hospital index.

Calculating marginal effect sizes from multivariate regressions in Stata/BE version 17.0 between June 6, 2022, and September 23, 2022, we tested associations between caseloads with Black and Hispanic populations and Disproportionate Share Hospital index and the receipt of penalties, controlling for hospital characteristics and case mix. Two-tailed *P* < .05 was considered statistically significant. The Washington University Human Research Protection Office approved this cross-sectional study, which followed the STROBE reporting guideline. Informed consent was waived due to the deidentified nature of the data.

## Results

We identified 735 unique hospitals in CJR between 2016 and 2019, contributing 2161 hospital-years of participation. The highest annual participation was in 2017, with 702 hospitals, while the lowest annual participation was in 2018, with 389 hospitals (Table). The percentage of mandatory participant hospitals penalized increased yearly (Figure). In 2017, 23.1% (162) of mandatory participants were penalized, including 39.3% (5) of safety-net hospitals and 41.4% (58) of hospitals with high Black and Hispanic populations. After low-cost hospitals were allowed to exit in 2018, 44.5% (173) of hospitals whose participation remained mandatory were penalized, including 69.7% (62) of safety-net hospitals and 64.4% (58) of hospitals with high Black and Hispanic populations. In 2019, 52.8% (209) of mandatory participants were penalized, including 87.9% (80) of safety-net hospitals and 71.7% (66) of hospitals with high Black and Hispanic populations.

**Table.** Characteristics of Hospitals Mandated to Participate in CJR and Marginal Effect Sizes for Penalization Status

Variable	Participation characteristics, No. (%)^a^				Marginal effect size for penalization status (95% CI)^b^		
	2016	2017	2018	2019	2017	2018	2019
Total	674	702	389	396	NA	NA	NA
Penalized	0	162 (23.1)	173 (44.5)	209 (52.8)	NA	NA	NA
Teaching status							
Nonteaching	254 (37.7)	267 (38.0)	136 (35.0)	141 (35.6)	0 [Reference]	0 [Reference]	0 [Reference]
Teaching	420 (62.3)	435 (62.0)	253 (65.0)	255 (64.4)	−0.10 (−0.17 to −0.02)	0.06 (−0.06 to 0.17)	0.07 (−0.04 to 0.18)
Size							
Small	227 (33.7)	239 (34.0)	120 (30.8)	119 (30.0)	0 [Reference]	0 [Reference]	0 [Reference]
Medium	230 (34.1)	246 (35.0)	138 (35.5)	144 (36.4)	−0.07 (−0.15 to 0.01)	0.13 (0.01 to 0.25)	0.14 (0.03 to 0.24)
Large	217 (32.2)	217 (30.9)	131 (33.7)	133 (33.6)	−0.06 (−0.15 to 0.03)	0.10 (−0.04 to 0.23)	0.16 (0.03 to 0.29)
Ownership							
Public	90 (13.4)	92 (13.1)	37 (9.5)	37 (9.3)	−0.07 (−0.19 to 0.05)	−0.30 (−0.48 to −0.12)	−0.09 (−0.26 to 0.08)
Nonprofit	423 (62.8)	443 (63.1)	245 (63.0)	250 (63.1)	−0.13 (−0.22 to −0.03)	−0.27 (−0.40 to −0.15)	−0.34 (−0.44 to −0.23)
Private	161 (23.9)	167 (23.8)	107 (27.5)	109 (27.5)	0 [Reference]	0 [Reference]	0 [Reference]
Geographic location							
Northeast	143 (21.2)	147 (20.9)	131 (33.7)	132 (33.3)	0 [Reference]	0 [Reference]	0 [Reference]
Midwest	141 (20.9)	146 (20.8)	26 (6.7)	26 (6.6)	0.08 (−0.02 to 0.18)	0.02 (−0.17 to 0.20)	−0.05 (−0.23 to 0.14)
South	207 (30.7)	215 (30.6)	165 (42.4)	168 (42.4)	−0.03 (−0.12 to 0.06)	−0.04 (−0.15 to 0.08)	−0.06 (−0.17 to 0.04)
West	183 (27.2)	194 (27.6)	67 (17.2)	70 (17.7)	0.00 (−0.09 to 0.09)	0.09 (−0.06 to 0.25)	0.25 (0.13 to 0.37)
Rurality							
Nonrural	650 (96.4)	641 (91.3)	363 (93.3)	370 (93.4)	0 [Reference]	0 [Reference]	0 [Reference]
Rural	24 (3.6)	61 (8.7)	26 (6.7)	26 (6.6)	−0.03 (−0.14 to 0.08)	−0.04 (−0.26 to 0.19)	−0.11 (−0.26 to 0.03)
Race and ethnicity							
Lower proportion of Black and Hispanic patients	540 (80.1)	562 (80.1)	299 (76.9)	304 (76.8)	0 [Reference]	0 [Reference]	0 [Reference]
High proportion of Black and Hispanic patients^c^	134 (19.9)	140 (19.9)	90 (23.1)	92 (23.2)	0.36 (0.25 to 0.46)	0.33 (0.19 to 0.49)	0.32 (0.17 to 0.47)
Safety-net hospital status							
Non–safety-net hospital	540 (80.1)	562 (80.1)	300 (77.1)	305 (77.0)	0 [Reference]	0 [Reference]	0 [Reference]
Safety-net hospital	134 (19.9)	140 (19.9)	89 (22.9)	91 (23.0)	0.34 (0.22 to 0.46)	0.33 (0.16 to 0.50)	0.42 (0.27 to 0.57)

Abbreviations: CJR, Comprehensive Care for Joint Replacement Model; NA, not applicable.

^a^ Data are expressed as the number (percentage) of hospitals in the cohort. For example, in 2017, 435 teaching hospitals were mandated to participate (comprising 62.0% of mandatory participants in 2017). In 2018, 253 teaching hospitals were mandated to participate (comprising 65.0% of mandatory participants in 2018).

^b^ Data are expressed as the marginal effect size (95% CI) of each hospital characteristic on penalization status, relative to the characteristic’s reference group.

^c^ Race and ethnicity were defined according to beneficiaries’ self-selected race and ethnicity at the time of Medicare enrollment.

**Figure.**
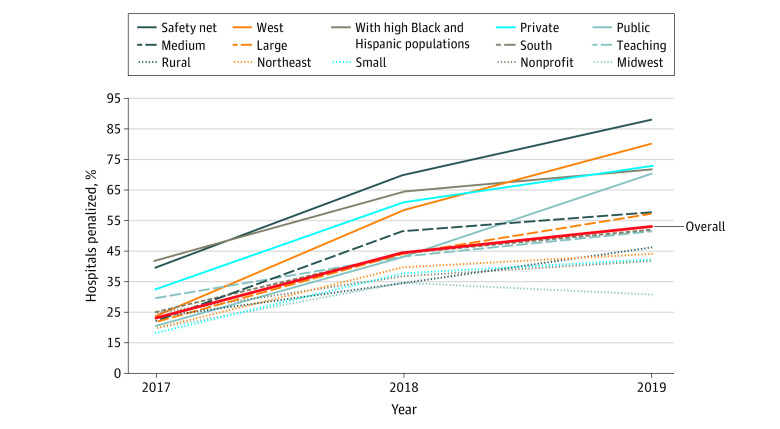
Proportion of Mandatory-Participation Hospitals Receiving Penalties Under Comprehensive Care for Joint Replacement (CJR) Model, by Hospital Characteristics By design, CJR did not levy penalties in 2016. For each characteristic, data are plotted as the percentage of hospitals penalized relative to the number of hospitals that were mandated to participate that year. For example, 20.5% of teaching hospitals that were mandated to participate in 2017 were penalized, and 51.2% of teaching hospitals that were mandated to participate in 2019 were penalized.

Controlling for hospital characteristics and case mix, hospitals with high Black and Hispanic populations (marginal effect size, 0.32; [95% CI, 0.17-0.47]; *P* < .001) and safety-net hospitals (marginal effect size, 0.42; [95% CI, 0.27-0.57]; *P* < .001) that were mandated to participate in 2019 had higher probabilities of being penalized than hospitals with lower Black and Hispanic populations and non–safety-net hospitals (Table).

## Discussion

Differences in Medicare’s allocation of penalties were large and increased after 2 overlapping policy changes in ways that have implications for equity. First, Medicare allowed hospitals in lower-spending areas to exit CJR after 2017; hospitals mandated to participate thereafter were penalized at higher rates. Second, Medicare lowered spending targets for high-risk hospitals by shifting from hospital-specific targets to multihospital shared targets that did not account for differences in medical or social case mix between hospitals.^1,4^ These lower spending benchmarks may have been less attainable for safety-net hospitals and hospitals with high Black and Hispanic populations, in part because they serve patients who have greater needs engendered by systemic barriers to care and thus remain persistently high spending.^5,6^

This study has limitations. The 2019 penalties are preliminary, although historically less than 5% of preliminary penalties become bonuses on finalization. In 2021, Medicare began adjusting benchmarks for patients’ medical and social complexity; performance data after 2019 are not available due to COVID-19-related delays.^1^

In this cross-sectional study, differences in the types of hospitals penalized under CJR were found to have widened. Regional benchmarks in CJR may inadvertently serve an institutionalized function of penalizing the safety-net hospitals.^2^ Policy makers should ensure that hospitals receive achievable spending benchmarks to avoid widening disparities in care.
